# The determinants of vaccination in a semi-rural area of Vientiane City, Lao People’s Democratic Republic: a qualitative study

**DOI:** 10.1186/s12961-018-0407-9

**Published:** 2019-01-09

**Authors:** Vanphanom Sychareun, Lucy Rowlands, Phoutsomphong Vilay, Jo Durham, Alison Morgan

**Affiliations:** 1 Nossal Institute for Global Health, Melbourne School of Population and Global Health, University of Melbourne, 5th Floor, 333 Exhibition St, Melbourne, 3000 Australia; 2 Faculty of Postgraduate Studies, University of Health Sciences, Vinetiane, Lao PDR; 3 School of Public Health and Social Work, Queensland University of Technology , Brisbane, Australia

**Keywords:** Vaccination, immunisation, Extended Plan of Immunisation, supply, demand

## Abstract

**Background:**

Immunisation is a cost-effective and highly efficacious public health intervention, saving over 20 million lives in the last two decades due to decreases in childhood bacterial infections. In the Lao People’s Democratic Republic, significant gaps in childhood immunisation coverage rates remain, which are a cause for concern and a barrier to the country reaching its Sustainable Development Goal targets for child health. Efforts to increase coverage have had limited success, with widening inequities being observed between urban and remote and rural areas.

**Methods:**

The objectives of this study were two-fold; firstly, to describe the knowledge, attitudes and practices of mothers regarding their children’s immunisation status; and, secondly, to identify individual and health system determinants of access to immunisation in five rural villages within a rural district in Lao People’s Democratic Republic. This qualitative research used observation and interviews with healthcare workers (*n* = 10) and mothers (*n* = 10) with at least one child aged 12–23 months.

**Results:**

The study identified several health system barriers that lower community demand for immunisation. These included the use of multiple providers, inconsistent record keeping and an inadequate health information system. At the individual and household level, there was a lack of understanding of the role of immunisation and the role of the different services provided.

**Conclusions:**

The study suggests that increasing immunisation coverage in Lao People’s Democratic Republic requires clearer immunisation pathways, an integrated or unified information recording system across the different levels of the health system, and strategies to increase demand, including increasing individual and household understanding of the role of immunisation in child health.

**Electronic supplementary material:**

The online version of this article (10.1186/s12961-018-0407-9) contains supplementary material, which is available to authorized users.

## Background

Early childhood vaccination is an essential, cost-effective public health measure to reduce preventable premature child mortality and childhood morbidity [1–3] and contributes to lowering the spread of vaccine-preventable diseases [4, 5]. Recognising the public health importance of immunisation, WHO launched its Expanded Programme on Immunization (EPI) in 1974, recommending a standard immunisation schedule covering tuberculosis, Bacillus Calmette–Guérin (BCG), polio, diphtheria, tetanus, pertussis, and measles [3]. However, despite the public health benefits of immunisation, WHO estimates that based on the standard measure of the proportion of children who receive the full series of three doses of diphtheria-tetanus-pertussis vaccines by 12 months of age, over 19 million infants per year do not receive all of the recommended basic vaccines [1]. Increasing immunisation rates, particularly in lower- and middle-income countries where the burden of vaccine-preventable disease is highest, is an ongoing public health effort [6], prioritised by international agreements including the Sustainable Development Goals and the Global Vaccine Action Plan 2011 to 2020 [5].

Reasons for non-vaccination relate to both the demand and supply-side determinants and include access to immunisation services and parents or caregivers understanding of benefits of vaccination compared to the risks, when to get their children vaccinated and sociocultural norms [6–10]. Concerns about the safety and efficacy of vaccines and trust in the government and medical professionals has also been found to influence vaccine acceptance [9–11], with individuals positioned along a continuum of complete acceptance to complete refusal [10]. In lower- and middle-income countries, determinants of immunisation have been associated with parental education [12–14], general health knowledge [14], religious beliefs [15, 16] and rurality [17]. Access to healthcare services, including antenatal care (ANC) and facility-based birthing, as well as logistical challenges in reaching healthcare services, have also been found to be determinants of child vaccination [11, 17, 18].

Situated in South-East Asia, the Lao People’s Democratic Republic is an ethnically diverse and mountainous country with a population of 6,492,228, based on the most recent census [19]. The country is a low- to middle-income country, in the process of rapid economic development characterised by an average annual gross domestic product growth rate of 8% over the past decade, and a halving of the national poverty rate [20]. Nevertheless, despite economic growth and improvement in many social indicators, inequities are increasing with disparities in poverty reduction and access to health and other basic services. Living in a rural area, being from an ethnic minority group or having a low level of education are particular markers of disadvantage in health outcomes [21].

As with other indicators of social progress, the country has made significant progress in reducing maternal mortality, but at an estimated 197 per 100,000 live births (80% uncertainty interval 136–307), maternal mortality is one of the highest in the region [22]. The under-five mortality rate has also improved, reducing from 68 deaths per 1000 live births in 2012 [22] to 46 in 2017 [21]. While positive, improvements are uneven and likely to be due to general socioeconomic development, especially in urban areas, increased access to skilled birth attendants and health services including immunisation, and enhanced breastfeeding practices [20]. Achieving the Sustainable Development Goal targets in relation to reducing under-five mortality requires increased investment in evidence-based, high-impact interventions targeted at women and their families in rural areas [20].

Childhood immunisation is a proven intervention that can protect against several communicable diseases and morbidity and mortality. Expanding coverage of the childhood immunisation programme has been a priority for the Government of the Lao People’s Democratic Republic. Poliomyelitis was eliminated in 2000 and immunisation against measles and neonatal tetanus has also increased [21]. New vaccines for the prevention of viral hepatitis, bacterial meningitis and pneumonia have also been added to the national immunisation schedule [23], which involves five contacts with the child (Table 1). Immunisation coverage is currently at 48% of children 12 to 23 months being fully immunised; however, this is substantially below the government’s target of 90% and is inequitable, with wealthier groups and urban families having higher levels of coverage [20]. Furthermore, while overall coverage has increased since 2006, changes in the equity gap have plateaued [21].

**Table 1:** EPI schedule

Contact	Immunisation	Schedule
1	Hep B BCG	< 7 days old 0–11 months
2	Pentavalent: diphtheria, tetanus, pertussis, Hep B, haemophilus influenza type B	2, 4, 6 months
3	Oral poliovirus vaccine	2, 4, 6, 18 months
4	Pneumococcal conjugate vaccine	2, 4, 6 moths
5	Measles	12 months

In rural areas, two of the main reasons for low immunisation coverage are low uptake of facility-based birthing and high dropout rates. Some of the underlying reasons relate to demand and supply-side barriers, including the quality of immunisation services and parental knowledge and attitudes. Rural families often have limited access to reliable services and often experience negative health staff attitudes. Other supply-side failures include delays in disbursement of funds, vaccine shortages, and poor cold chain and data management [24]. Even in urban areas where there is higher uptake of facility-based birthing, a serological study found only 6.6% of infants had protective anti-hepatitis B antibodies [25]. The most recent Lao Social Indicator Survey reported vaccine coverage rates of 82% for BCG, 73% for Penta 1 (diphtheria, pertussis, tetanus, hepatitis B and Hib), 67% for Penta 3, 48% for pneumococcal conjugate vaccine 3, 69% for oral polio vaccine 3 and 66% for measles [21]. Late or incomplete vaccinations have contributed to outbreaks of pertussis and diphtheria across the country [23]. To address the gap in immunisation coverage, the Lao government is currently preparing legislation that will require children and expectant mothers to have all required vaccinations on the immunisation schedule. Effective implementation of this law requires an understanding of the supply and demand side determinants of immunisation coverage.

The purpose of this study was to examine the supply and demand side determinants that promote and constrain immunisation coverage in five villages of Sangthong District, in the Lao People’s Democratic Republic for mothers of children aged 12–23 months. More specifically, the study objectives were to describe the knowledge, attitudes and practices of mothers regarding their children’s immunisation status and identify individual and health system determinants of access to immunisation. While studies on the determinants of certain behaviours often use quantitative approaches, studies on the determinants of immunisation have frequently been qualitative [11]. Given our research questions and that our research was exploratory, we also selected qualitative methods to enable a more in-depth nuanced understanding of the determinants of immunisation [11].

## Methods

As an exploratory study, this was a qualitative research design that used observation, interviews with healthcare workers (HCWs) and mothers with at least one child aged 12–23 months. This study design was chosen to explore, in depth, the reasons for supply- and demand-side failures in increasing immunisation coverage. In addition, a health audit was undertaken to collect data on immunisation coverage. The audit involved recording general information about the facility, the staff, vaccination stock and equipment, and provided important background information to the study.

### Study sites

The study was undertaken in Sangthong District, Vientiane Capital City. The Capital City has nine districts and a population of nearly 900,000 inhabitants. Sangthong District, one of the poorest districts in the province, was selected because it has a staffed health facility that delivers immunisation services and a significant proportion of ethnic monitory groups. A cluster of five villages surrounding the health centre (HC) was selected based on the subjective assessments of key informants and local experts, based on villages they felt would provide information-rich data and where healthcare staff had expressed an interest in understanding more about the barriers to immunisation uptake in this area. Each village was approximately a 2-hour drive from the capital in the dry season. Staff at the HC and leaders in each of the selected villages were contacted in advance by the health district personnel, were advised about the nature of the study and their cooperation was sought.

### Study participants

Participants were mothers with at least one child aged 12–23 months and HCWs. In each village, a purposive sampling framework was prepared to help identify potential participants who were mothers of partially immunised children and mothers of children who were fully immunised according to the EPI schedule. Immunisation status of children was established by reviewing immunisation cards held by the women, when available. In cases where the card was lost or not available, the lead researcher (LR) asked the mother to recall their child’s immunisation history from memory and the child was also checked for a BCG scar.

Mothers were identified using two recruitment strategies. The first was through the assistance of a district health nurse who introduced the field researcher (LR) to village health volunteers and traditional birth attendants in each village, who then nominated potential mothers to be invited to participate based on women who would potentially provide a rich source of data. The second recruitment strategy identified and invited eligible mothers to participate during the immunisation campaign after immunisations and supplements were given. HCWs from all four levels of the health system, who were involved in the promotion and delivery of immunisation to each of the villages, were invited to participate. Recruitment was via direct invitation to HCWs at each level and included a representative of a non-government organisation involved in district health services. In total, 10 in-depth interviews with healthcare providers and 10 with mothers were conducted. Recruitment was restricted to two mothers per village, according to the availability of the mothers who needed to return to work as well as our inclusion criteria.

### Data collection

The lead researcher (LR) and the Lao-based researcher (PV) were observers taking notes as they observed education sessions and implementation of the immunisation campaign in the villages. This allowed the researchers to gain insight into how the community health outreach workers and women engaged during immunisation outreach activities and capture the context within which the different actors interacted [26, 27]. The health audit and HCW interviews conducted at the HC provided background information to the study. The audit included reviewing HC documents including, where available, the monthly log books where immunisations should be recorded and facility observation. While we wanted to obtain coverage data from the health facilities, this was not possible as the log books were not always available or were incomplete, meaning we could not obtain reliable quantitative data from these sources. Similarly, at the village level, it was not possible to obtain coverage data as immunisation cards were often missing.

Face-to-face, in-depth, semi-structured interviews were undertaken with participating mothers (*n* = 10) and HCWs (*n* = 10), with a trained bilingual research assistant (PV) taking detailed notes during the interviews. The use of semi-structured interviews allowed the researchers to cover all relevant areas of interest, while still allowing some spontaneity and flexibility so that participants could raise issues of interest to them, enriching data collection [28, 29]. The guideline used for interviewing the mothers included sociodemographic information, immunisation status of their child, knowledge, attitudes and practices towards infant immunisation, and barriers to achieving full immunisation (Additional file 1). In addition, using visual pictures of vaccines and disease cards, mothers were asked to match three vaccines (polio, measles and BCG) with the disease they prevent. The interview guide for the HCWs included general information (date/time/position of healthcare provider), information on immunisation coverage, vaccination equipment and practice, procurement and vaccine distribution barriers to the programme implementation, as well as the organisation, management and funding of immunisation services. The interviews also allowed the researchers to follow-up and clarify findings from observations and the health audit.

Prior to participating in the study, potential study participants were read a plain language statement which outlined study purpose and the voluntary nature of participation and informed consent obtained. Interviews were conducted either in English or Lao depending on participants’ preference and in a location convenient to participants. Interviews were recorded with permission and transcribed by the Lao research assistant while still in the field. As the transcripts and observational notes were written up daily by the field researchers (LR and PV), they were able to discuss preliminary results and thus respond to emerging themes in successive interviews.

### Data analysis

All transcribed data was read multiple times to identify key codes and themes and capture initial meanings and patterns as they related to factors that support or deter vaccine uptake. Observational and interview data was manually coded independently by the lead investigator (LR) and guided by the research objectives [28, 29]. The codes were merged into larger categories and themes guided by the themes outlined a priori in Table 2. These themes were identified based on our understanding of the literature and our practical experience of working in Lao People’s Democratic Republic. While themes were identified a priori, this did not prevent other themes emerging [26, 27]. Throughout the analytic process, there was a moving back and forth between the entire dataset and the coded extracts, with interpretations checked with the research team and continuous analysis throughout the writing process [30].

**Table 2:** Framework use for data analysis

Objective 1: Health systems and supply-related determinants of immunisation coverage	Issues with recording and documentation of coverage by healthcare workers Human resources Vaccine quality and supply
Objective 2: Service utilisation and community demand-related determinants of immunisation coverage	Community knowledge Poor knowledge of effectiveness and action of immunisation Uncertainty with campaign knowledge Multiple and unclear education sources Community attitudes Inadequate handling of immunisation card Being afraid of side effects High satisfaction with service Preference for fixed site provider Access determinants Distance between health facility and village making it difficult to access services Lack of time to access services – rice fields and household duties Lack of money deterring utilisation Seasonal variation in utilisation and ease of access

### Ethical clearance

The study was approved by the research ethical committee of the University of Health Sciences, Lao People’s Democratic Republic, and the Human Research Ethics Committee of the University of Melbourne. Privacy and confidentiality were assured for the key informants to participate in this study.

## Results

### Sociodemographic characteristics

The age of the interviewed mothers ranged from 20 to 35 years old and each had one to three children aged 12–23 months. Travel time from their village to the HC differed, with the closest being a 1-minute walk, and the furthest 40 minutes walk. Table 3 outlines the characteristics of the mothers included in the study and the immunisation status of their children.

**Table 3:** Sociodemographic characteristics of mothers aged 20 to 35 years old with children aged 12–23 months, and their immunisation status

No.	Age	Ethnicity	Location from nearest HC	No. of children	Immunisation card available? Yes/No	Immunisation status of child	Comment
Incomplete Immunisation							
1	22	Lao Lum	40 min walk 5 min motorbike	2	No – Lost	Incomplete	BCG scar present Mothers recall: Missing measles, only received one OPV, unsure about others
2	35	Lao Lum	30 min walk	1	Yes	Incomplete	Missing Hep B birth dose, measles, OPV1 and OPV3
3	25	Lao Lum	30 min walk 15 min motorbike	1	Yes	Incomplete	Missing Hep B birth dose Different immunisation card – French
4	23	Lao Lum	20 min motorbike	2	Yes	Incomplete	Missing Hep B birth dose
5	25	Lao Lum	5 min motorbike	2	No – Lost	Unclear – most likely incomplete	Mother recall: “*5 injections – leg 2 times and arm 3 times*” and oral drops “*two times*” Most likely missing one DTP-hep and one OPV
6	30	Lao Lum	1 min walk	3	No – Lost	Unclear – most likely incomplete	BCG scar present Mothers recall: ‘*1 time in leg*” and “*3 times oral drops*” Most likely missing two DTP-hep doses. Possibly missing measles and/or Hep B birth dose
	Complete Immunisation						
5	20	Lao Lum	2 min motorbike	1	Yes	Complete	
6	28	Lao Lum	30 min walk	1	Yes	Complete	
7	28	Lao Lum	5 min motorbike	2	Yes	Complete	
10	21	Lao Lum	5 min walk	1	Yes	Complete	

*BCG* Bacillus Calmette–Guérin, *DTP* diphtheria, tetanus, pertussis and haemophilus influenza type b, *Hep B* hepatitis B, *OPV* oral polio vaccine

### Determinants of immunisation coverage

The interviews helped to identify factors that improve immunisation coverage as well as barriers to increasing immunisation coverage. Two main themes were identified, namely (1) health system- and supply-related factors, and (2) service utilisation and community demand factors (Table 2).

### Health system- and supply-related factors

#### Recording and documentation of coverage

Incomplete and inaccurate documentation emerged as a key issue. None of the HCs, village heads or village health volunteers (VHVs) could provide quantitative data on the level of coverage at the village level, making it impossible to estimate coverage. While each village HC should record immunisations in a separate book, with the same book used to record community outreach immunisations, records were incomplete. Furthermore, according to some HCWs, the book was not always taken during community activities. While on the day of the immunisation campaign some mothers brought immunisation cards along with them, they were frequently reported as lost or misplaced. According to HCWs, another reason why some mothers may not have had immunisation cards is because sometimes the HC has insufficient cards for the population and that this could last several months. Interviews with mothers and HC staff also revealed that household mobility and provider preferences, with mothers not consistently using the same provider, also compromised the quality of the available immunisation data as there is no centralised data storage system. As one HCW explained:

“*People move from one district to another and it’s hard to record the immunisations a child has had…There is poor communication between provinces, districts and health centre.*” (HCW 1)

With no central or district level information system where patient and immunisation records are kept and shared, HC staff said it was almost impossible for the HC to track families moving in or out of villages. In moving, families rarely brought their immunisation cards with them and frequently could not remember the last immunisation or how many doses a child had received.

Household movement, lost immunisation cards, gaps in HC recording and the lack of a centralised reporting system where data is shared also meant that it was possible that some children received more than the recommended doses, especially during community outreach activities when VHVs tend to gather all the village children under the age of five for immunisation, regardless of their immunisation status. As one HCW explained:

“*If the village health volunteer had a book with coverage they would know what’s going on and could tell the health staff which children to target when they visit during outreach activities and would know what child needs what injection. But at the moment the volunteer doesn’t know who has had what immunisation! The volunteer knows her village people… ‘that boy’, ‘that girl’ – but the health staff don’t know people like that.*” (HCW 7)

Poor data management at the village also means the HC immunisation coverage data submitted weekly to the district level, where it is compiled and sent to the provincial level and then forwarded to the National EPI centre, is likely to be incomplete.

### Human resources

#### Salary and incentives

The HC staff generally demonstrated a positive attitude towards their work, stating that they felt appreciated by their community and proud of their role. However, all HC staff interviewed felt their salary and the incentives were insufficient to cover living costs, meaning they often undertook additional private work, sometimes while they should have been working in the HC. Furthermore, salaries were not always paid on time, with one person explaining that, in the previous year, salaries were paid 3 months late.

Similarly, VHVs, who do not receive a salary, and HC staff felt the allowance of 50,000 kip per day (USD 6) provided for training in the district HC 2 days every 3 months, was insufficient to cover travel, accommodation and the opportunity costs of not working in their rice-fields for 2 days. The lack of compensation for accommodation meant that, rather than working all day on immunisation campaigns, staff sometimes left early to travel home each evening. As one VHV explained:

“*I don’t stay the night there – I go to District Hospital then back to my village in the evening after dark because the money is not enough to pay for a room to stay. It’s a 50 min walk.*” (HCW 3)

The HC staff also complained that they often had to use their own transport for community outreach activities. Women also said they found it hard to maintain balance when riding their motorcycle while also carrying the cold box.

#### Adequate staffing

Within Sangthong District there are seven HCs, yet the uneven distribution of staff, with most working closer to the district centre, meant only three of the seven were fully operational. Inadequate staffing resulted in the HCs opening at inconsistent times, creating a barrier to access for busy mothers, as one mother explained:

“*We need the village doctor* [healthcare worker] *to work regularly… because sometimes it’s open and other times its closed and we don’t know. Sometimes they work in the morning and take the afternoon off.*” (Mother 2)

Inadequate staffing during community outreach activities meant mothers often had to wait for long periods of time for their child’s immunisation, with some leaving without getting their child vaccinated. A common sentiment expressed by the VHVs illustrates this:

“*We need more vaccinators… Because we only have one vaccinator at health centre and if he is sick… children can’t get the vaccine sometimes. We need more staff so when mother goes to get the injection she can get it for her child.*” (HCW 3)

This view was also supported by HC staff who also commented on the uneven distribution of staff. Salaries are the same at the district level; however, working at the district level is more convenient and offers better access to other services than at the village level for the HCWs.

#### Training and supervision

While training was provided to the VHVs every 3 months, there was no evidence of a curriculum or schedule of the content covered. Furthermore, according to one of the VHVs, the last immunisation training he had received was 1 year previously. One of the reasons given for infrequent training was the low number of staff at the district level and limited support from the provincial level. However, most of the HCWs expressed a desire to further develop their skills. One stated*:* “*I want to train so I can protect my people*” (HCW 3). Another respondent reported: “*I want to train in how to give injections, so I can help outreach when they come to my village*” (HCW 4).

In the observed immunisation campaign, EPI supervisors from the district and HC were allocated to each village for outreach activities. A number of staff, however, including the Head of the EPI department, felt supervision was low, partly due to lack of staffing and funding, as the following two quotes help to illustrate:

“*I observed at District Hospital for a couple of days before the last immunisation campaign, and the staff were not prepared, they were all rushing around at the last minute. Government staff is lazy, they are not strict, and they depend on the district head of immunisation and this person doesn’t have a schedule or checklist either to tell them what to do leading up to the campaign, so they don’t plan.*” (HCW 7)

“*They only stay half a day in the village then go back…farmers complain that the health workers don’t stay all day for outreach…immunisation has no supervisor in the field so people in the capital don’t know if the health worker only stays half a day and so they get away with only doing half a day’s work but getting the full pay.*” (HCW 7)

“*In the past the supervisor from the EPI centre in Vientiane came to the district hospital to advise and follow-up the immunisation programme three or four times per year… now the supervisor only comes once a year.*” (HCW 1)

One HCW felt that the supervisor should come from the funder (the NGO), “*so they can see what is truly going on*” (HCW 7).

### Vaccine supply

At the time of the study, observations and interviews suggested that there were no shortages regarding vaccine equipment, syringes, refrigerators, sharps disposal boxes, cold boxes, sterilised wipes and rubber gloves. However, according to health staff, approximately twice a year there were stock-outs of vaccines for 2 to 3 weeks. According to the health staff interviewed, sometimes, when preparing for community outreach they had insufficient vaccines and would have to try and obtain additional supplies from neighbouring districts. The head of the EPI, however, stated vaccine outages had decreased with community outreach having sufficient stock in recent campaigns.

Many of the HCWs also said that they did not get all the doses they had ordered, as one explained: “*If I put in for 30 doses, I get 10, or if I put in for 100 doses, I usually get about 50*” (HCW 5).

### Service utilisation and community demand-related issues

#### Community knowledge

Using visual vaccines and diseases cards, mothers were asked to match three vaccines (polio, measles and BCG) with the disease they prevent. The results (Table 4) suggest that mothers had limited knowledge, with only two out of ten matching all three correctly. Mothers were also asked if they knew at what age the same three vaccines were given. Four mothers correctly identified measles as being given at 9 months; however, all other answers were incorrect. Mother’s knowledge about immunisation was low and generally restricted to understanding in a general way as being “*…important*” in preventing disease (Mother 1) and “*…stops disease*” (Mother 8). Five mothers recalled being told by HCWs that it was ‘good’ but were unaware of the benefits of vaccination*.* Mothers also held deferential attitudes towards HCWs, and were reluctant to ask them questions as the following interview excerpts highlight:

“*I am told to get the immunisation, but I don’t know why I should get it or what it is they are giving my son. I don’t want to ask, but inside I want to know.*” (Mother 2)

**Table 4:** Mother’s exercise 1 Results – ‘Matching Vaccine with Disease’

Exercise 1 results	Number of mothers
Matched all three correctly	2
Matched one correctly	5
Matched none correctly	3

“*I know where and when to get injection, but not why I must get it and why it is good for the child*”*.* (Mother 3)

Few of the mothers interviewed understood the need for immunisation in the first few months of a child’s life.

“*I know where are when to get injection but not why I must get it and why it is good for the child*”*.* (Mother 3)

This low level of understanding contrasts with opinions of HCWs, who generally considered that mothers had a good understanding of the benefits of immunisation. As one HCW noted, for example,

“*Mother’s knowledge is no problem now – they understand about immunisation*”(HCW 1).

Mothers learned about the EPI campaigns via a range of means. The most common ways, however, were the VHV going house to house the night before and a village alarm declaring the campaign early the next morning. Nevertheless, the short notice meant that the mothers could not always attend. As one mother explained:

“*It would be better if they told us more times, and with some more time before they come so we can plan ahead who goes to work, who will go to the rice field and who will take the baby to get the vaccine. Because the village volunteer tells me the night before and the next morning they come, and sometimes the mother isn’t home, so they don’t know!*” (Mother 1)

Misunderstandings regarding who should present for immunisation during community outreach were also evident. According to the mothers and the HCWs, all children in the village at the time of the campaign are gathered without checking their immunisation status, as one HCWs stated:

“*They* [healthcare workers] t*ell everyone to come to outreach but don’t explain people who should come, so the mothers bring their child to get the vaccine but maybe they don’t need it; and then they say, ‘why doesn’t my child get the vaccine?’ – and then mothers get a bad feeling*”*.* (HCW 7)

#### Knowledge sources

Most of the mothers heard about child immunisation from a range of HCWs. Some were aware of community meetings in which information about immunisation was disseminated but could not remember what they had been told. Furthermore, the mothers themselves did not always attend the meetings, with another family member going in their place. However, this person did not always remember the information or share the information with the rest of the family, as the following interview extract helps to exemplify:

“*The doctor came to teach about health one time, but I didn’t go because in my village, usually the head of family goes and so my grandmother goes. Afterwards my grandmother is supposed to report back to the family, but she doesn’t because she does not understand what the doctor says*”*.* (Mother 4)

#### Community attitudes

##### Importance of immunisation

All 10 mothers stated immunisation was ‘very important’ to them, despite having limited knowledge of its benefits or the number of doses required. Three of the 10 mothers had lost their child’s immunisation card.

##### Side effects

All the mothers reported being concerned about potential side-effects of immunisation, particularly when their child or someone they knew had been negatively affected. As one mother explained:

“*When I went to get the first vaccine, my son got sick and I was so worried about his fever so I was afraid of going back again*.” (Mother 2)

The HCWs also recognised this, as the following quote illustrates:

“*Some mothers take their baby away when the doctors come to the village. They don’t want their child to get the vaccine because the parents are afraid the injection will make the baby sick…This mother in my village took her baby away when outreach came, so I door knocked and ask her to take the child to the health centre to get the injection… but the next time outreach came the mother didn’t go*” (HCW 3)

Mothers also expressed concern the needle would harm the baby or restrict the infant’s physical development. Most mothers said the wanted the HCWs to provide more explanation on the potential side-effects of vaccines and what to do if they occur, but even after knowing this, they were not sure they would be reassured. For the mothers, the best reassurance was seeing their child and others completing their immunisation with no side-effects. On the other hand, some of the mothers wanted signs that the immunisation had ‘worked’, as one explained:

“*The first time my baby got an injection there was no reaction and no scar, and I was scared it wouldn’t stop her from getting the disease, so I told the village doctor to inject her again. And so, he did, and the second time there was a reaction and a scar like the other babies, so I knew it had worked.*” (Mother 3)

### Preference of health service – fixed site compared to outreach

All mothers reported accessing immunisation services from multiple providers. Most preferred to use the district healthcare services and ANC rather than the outreach clinic. This was partly due to a generally higher level of trust in the capacity and technical know-how of the staff at the district level. While the community outreach clinics reduced the time and cost of travelling to the district, the downside was the time lost and inconvenience of waiting for the vaccination. For some mothers, the outreach service was more convenient. As one mother explained:

“*I don’t have a motor bike or car so it’s a problem for me. Sometimes I don’t have the money to pay for transportation, so I must borrow from someone to pay for the mini pick-up truck to the health centre. If I borrow a motor bike I must pay for the gasoline and this is expensive*”*.* (Mother 8)

In addition, as women often cannot drive, going to the district means having someone, usually their husband, to accompany them to the district HC. This means that two people are away from work for most of the day and physical access and workloads were identified as important reasons for not using district healthcare services, including for ANC and immunisation. On the other hand, community outreach teams were reported to often arrive late and not extend their outreach beyond the village centre. While some women left their child with the VHV all day, the inability of the community outreach to extend beyond the village centre can act as a barrier, especially during busy time in the fields, when women and their families may spend 3–4 months staying in their rice fields, which are often far from the village centre.

## Discussion

Protecting populations from vaccine-preventable diseases is shaped by interrelated supply and demand factors and actors that influence key immunisation programme outcomes such as coverage and completion of all vaccines at each of the aged-based WHO schedule points [31]. This research explored the supply and demand factors that promote and constrain immunisation coverage in Sangthong District in the Lao People’s Democratic Republic. Several supply-side factors were identified, some of which are not unique to immunisation but are also relevant to other primary healthcare interventions and have been reported elsewhere [18, 32, 33]. These include, for example, inadequate human resources, weak information systems, stock-outs and the capacity of district-level management for health [32–34]. Other factors included the community not always being informed of outreach dates or outreach sessions conflicting with other priorities such as livelihood and households’ obligations. Given supply-side factors affect demand, general health system strengthening is likely to be an effective strategy for achieving results [35]. Services must respond to individual and community needs in planning outreach and communities should be provided with opportunities to give input to service delivery decisions, including the timing of outreach activities. At the same time, the healthcare workforce needs to be given the autonomy and ability to adapt services to community needs.

While the HCWs generally reported that mothers and community knowledge about immunisation was ‘good’, the findings in the study suggested that mothers’ knowledge was very low and non-specific. This is of concern, as increased knowledge among families and communities has been found to be effective in increasing immunisation demand [34, 36]. A systematic review and meta-analysis, for example, found that, in low- and middle-income countries, education was likely to be more effective than incentives in increasing uptake of vaccinations, although both were effective [34]. Other studies in Lao People’s Democratic Republic have also suggested that poor immunisation knowledge among the Lao population is likely to negatively impact coverage [33, 37]. Studies in Lao People’s Democratic Republic, for example, have highlighted that mothers with awareness of the diseases targeted with immunisation and the correct number of required visits to the health facility had children with significantly higher immunisation rates [33, 38]. Also, noteworthy, is that mothers articulated a desire to better understand the reasons for immunisation and its links with disease prevention, expressing dissatisfaction with simply being told where and when the service could be accessed. Potentially, the health message has been oversimplified, with the HCWs overlooking the fact that mothers are the best carers for their children, and that they underestimate maternal ability to absorb complex information, or alternatively, they may lack the skills and resources to present complex ideas into readily understandable messages. Either way, current messages or delivery mechanisms do not seem to be meeting mother’s needs.

Currently, mothers and their families receive immunisation messages during community meetings or campaigns, in ANC visits, and from other family members or friends. A Cochrane review on the effectiveness of intervention strategies to increase and maintain high childhood immunisation coverage in lower middle-income countries found face-to-face interventions educating parents and other community members at village meetings would probably increase immunisation coverage [6]. The review also found that the use of lay or community health workers, such as the VHWs, to promote immunisation probably increases uptake [6]. Kaufman et al. [7, 39], on the other hand, found little or no improvement in immunisation coverage because of face-to-face communication. A systematic review evaluating the effectiveness of interventions designed to increase access to health services for children aged over 5 years in lower- and middle-income countries found that face-to-face education can address the knowledge gap demand-side barrier [18]. Overall, the evidence suggests that, in lower- and middle-income countries, community-based health education strategies will probably increase vaccine uptake and are likely to be more effective than facility-based health education [6]. However, it is likely that, to be effective, face-to-face communication needs to be tailored to the particular needs and concerns of mothers and their families [18] if financial and geographical issues that might act as barriers to immunisation are removed [32].

The tendency of mothers to access immunisation from several providers also potentially presents barriers to both the functioning of the health system and consumer knowledge. The HCWs felt that this ‘provider shopping’ was creating difficulties with coverage data recording and data sharing within the district. Provider shopping has been reported elsewhere in rural Lao People’s Democratic Republic, with few people using only one provider [40]. Not having a single entity responsible for delivering immunisation to the community may also result in a lack of responsibility for tracking and following up of children who are not recorded to be fully immunised, especially as vaccination records are often lost and the health information system is weak [41, 42]. This ‘smorgasbord’ of providers could be confusing for consumers, diluting the why, when and where of immunisation services. Some of the mothers, for example, seemed confused about immunisation accessibility and many were uncertain about whether they should wait for outreach at the village or seek immunisation elsewhere. Not knowing when the outreach teams were going to visit, or if they were coming when their child’s immunisation was scheduled, added to this uncertainty. This could be alleviated by household visits and provision of information before delivery, especially to families with lower levels of education [33]. With increasing access to mobile phone technology, text messages may also help to improve uptake of child health services, including immunisation [43].

A positive association has been identified between the possession of an immunisation card and immunisation status [36]. While the link is not clear, it could be that the presence of an immunisation card acts as a cue for the mother [36]. However, in this study several of the mothers did not have an immunisation card, highlighting the need for service providers to maintain a supply of cards, to ensure that these are provided to women and that women are educated on keeping the card in safe place and bring it with them when they take their infant to healthcare or community outreach services. Not being in possession of a vaccine card makes it hard for HCWs to determine an infant’s immunisation status, with oral report often accepted instead, but a poor correlation of verbal recall and serological immunity has been observed and may result in over-reporting [44]. Our results also suggest that HC data is either not available or incomplete, making it difficult to monitor and supervise immunisation services. To improve data management, HC staff needs further training and support in the execution of data management tasks.

he association between facility-based birthing and immunisation has been well-documented [36]. While not specifically examined in this study, demand for facility-based birthing in Lao People’s Democratic Republic and especially in rural areas remains low, and promoting ANC, facility-based birthing and postnatal care should continue to be a major strategy in improving child health [22]. Additionally, it is important to continue community outreach and promoting immunisation to mothers and their families given that a substantial proportion of women continue, for various reasons, to prefer giving birth at home [45, 46]. Another longer-term strategy to improve immunisation coverage is continuing to increase women’s literacy, including health literacy, through formal education but also through social networks [33, 47, 48]. Mother’s education, for example, has been positively correlated with immunisation; literate mothers, for example, may have more knowledge of immunisation, including target diseases and the immunisation schedule [33]. A study in India demonstrated a positive relationship between the proportion of literate females in a district and a child’s complete immunisation status within that district [47]. However, Cui and Gofin [48] found no association with mother’s education and child immunisation but noted that most of the participants in their study had low levels of education.

Another finding of this study is that a basic readiness to deliver immunisation services is important, but not necessarily enough on its own and investment is required in both supply and demand-side factors [34, 35]. While programmes to increase immunisation coverage have often focussed on supply-side factors, such as vaccine supply and cold chain management, and community-outreach to reduce geographical and financial barriers, some households were still missed as community-outreach teams did not go beyond the village centre. Provision of HCW training, including in ‘soft skills’ such as communication, may also help to address the supply-side barrier of acceptability and have indeed been effective elsewhere in increasing immunisation uptake [43]. Ultimately, both demand- and supply-side interventions are needed as increases in demand cannot be effective if supply-side constraints limit provision of vaccines [34, 49, 50].

As with all research, this study has some limitations that need to be acknowledged. Firstly, the study was conducted in one district, and is not representative of the country. Nevertheless, the study was not intended to be representative but was rather undertaken to qualitatively better understand persistent low immunisation coverage in Lao People’s Democratic Republic. Secondly, the interviews were undertaken using translation and inevitably some of the nuance has been lost and may have limited the depth of responses from participants. To mitigate this, a constant checking back and forth with interpreters was undertaken as well as triangulation of field notes, observations and interviews with community members and health staff. While we had initially wanted to estimate immunisation coverage based on health facility log-books, this was not possible due to missing data and monthly log books not always being retained. In addition, at the village level, in the absence of immunisation cards, the study also relied on recall. However, a population survey in Tanzania found parental recall of child immunisation was relatively good, being only slightly overestimated possibly due to social desirability bias, compared to a card-based checks [51].

To conclude, while impressive gains have been made in reducing infant mortality and increasing immunisation coverage, persistent inequalities remain. Increasing requires a focus on both supply and demand side factors, as well as broader socioeconomic intervention, including providing education to women and their families encouraging them to be active agents of their own and their children’s preventative health-seeking behaviour. Further research is also needed to better understand the pathways to improving immunisation uptake and how to build on strengths revealed in this study that may be supporting immunisation coverage, such as recent attempts to address supervision of HCWs in delivering immunisation and HCWs’ positive attitude towards helping their community. Further qualitative and quantitative work is also needed to better understand the interplay between the different supply- and demand-side factors and the relative importance of each. Comparison between districts with high immunisation coverage is also warranted to help to identify successful pathways to improving coverage.

## Additional file

Additional file 1: Interview guides for healthcare providers, mothers and health audit interview. (DOCX 692 kb)

## Abbreviations

ANC: antenatal care
BCG: Bacillus Calmette–Guérin
EPI: Extended Programme of Immunization
HC: health centre
HCW: healthcare worker
VHV: village health volunteer
