# Supplementary material for: The determinants of vaccination in a semi-rural area of Vientiane City, Lao People’s Democratic Republic: a qualitative study
Source: Health Res Policy Syst. 2019 Jan 9;17:2. doi: 10.1186/s12961-018-0407-9 (PMC6325875; doi:10.1186/s12961-018-0407-9)
Supplement: Supplementary file 1 — Interview guides for healthcare providers, mothers and health audit interview. (DOCX 692 kb) [file 12961_2018_407_MOESM1_ESM.docx]

# Appendix 1: HCW IDI Guide

The Determinants of Immunisation Coverage in Five Rural

Villages of Sangthong District, Lao People’s Democratic Republic

Thank you for agreeing to participate in this interview.

Do you have any questions before we start?

**1. General Information**

| Date |  |
| --- | --- |
| Time |  |
| Position of HCW at facility? |  |

**2. Immunisation Coverage**

In 2008, it was estimated that 73% of children in Lao PDR had received their first combined Diphtheria, pertussis and tetanus vaccine (DTP1).

- If this estimate truly represents the coverage levels among Lao PDR children.....

1. Why do you think some children receive the vaccines and some don’t?
2. What factors do you think make a child less likely to get vaccinated?
3. What factors do you think make a child more likely to get vaccinated?

- What happens when a child comes to the immunisation facility and is not fully vaccinated?
- Outreach –
  - Do you ever go outside the health centre to vaccinate? (if so, ask for details:

How? (transport)

Frequency?

How is the community notified?

- - Do you think that the health centre is accessible to everyone? If no ask whether they think this is a major barrier to coverage.
- Attitude of staff –
  - Do you think HCWs need to encourage mothers to attend? If yes, have you taken action to encourage them? If yes, please describe.
  - Do you feel as though you are appreciated in the community?
  - Do you feel as though you are adequately paid for your services?
  - Is your pay regular? (If no, ask how this makes them feel?)
  - Work: how long are your shifts? How many days a week do you work?
  - What is the attitude of other health workers you work with toward immunisation?
  - Do you think there are enough health workers at the centre to adequately deliver all the immunisations to children?
- Ordering –
  - Do you ever run out of vaccine?
  - Do you ever run out of syringes?

**3. Vaccination equipment and practice**

**Equipment**

- Adequate equipment –
  - Do you have enough equipment?
- Syringes –
  - What type of syringes do you use?
  - Have you been provided with auto disposable syringes?
  - Are new and clean syringes always available?

**Practice**

**Training:**

- When did you do your last immunisation training?
- What were the main areas this training covered?
- Local - Do you have a regular in-house in-service immunisation training within your Health Centre?
- Resources – Where do you keep training resources in your HC?
- Sharing: Do you usually share what you have learned with each other?
- Subject – What additional training do you heed to carry out your work, if any?
- Confidence to work- Do you feel confident delivering in immunisations?
- Confidence to train - After receiving training, have you ever taught others?
- Supervision – Are you supervised? Do you have a supervisory role?

**Multi-dose policy –**

- What is the multi dose policy?
- Are there times (e.g. under pressure) when the practice differs from policy?

If yes- can you give me an example of when this has happened?

- Is there any improvements you can suggest in the cold chain equipment and its management

**4. Procurement and vaccine distribution**

- Regular supply –
  - Has lack of vaccines ever stopped you vaccinating?
  - How often would you say this occurs?
- Is there any improvement you can suggest to help the facility have a regular supply of stock?

**5. Community issues**

- Target - Who attends your health centre?
- Does your health centre run any health education programs to promote attendance?
- Decision-makers - Which community members lead the community in health decisions?
- Drop-outs – Do you follow up drop-outs? If so, how often and how?
- Improvements - How could the service in your clinic be improved?
- Community involvement – Do you involve the community in your planning? If so, how often and how?

**6. Organization, management and funding**

- Could you suggest any improvements to the way your health facility is organised and managed?
- Do you feel as though your Health centre has adequate funds to provide immunisation services?
- Who is responsible for ensuring your Health Centre has enough funds to complete its planned activities?

**7. Barriers to Immunisation**

Now to finish off the interview, we have another game to play. Here on these cards we have some pictures of things that may stop a child from getting immunised.

Before I get you to play the game, I will explain the 6 cards:

1. This first card is TIME. It’s to do with mothers having enough time to get the immunisation.
2. The second card is DISTANCE: as you can see the health facility in this picture is far from the village, and it is hard for the mother to get the immunisation
3. The third card is AFRAID: mothers may be scared of immunisation hurting their baby, fever or side effects
4. The fourth card is to do with HCWs: there may not be enough to give the immunisation, or mothers may be scared of HCWs. OR maybe HCWs don’t give mothers enough information about immunisation
5. The fifth card is NO SERVICE: meaning that immunisations are not always available to mothers
6. The sixth card is POOR KNOWLEDGE. Maybe the mothers don’t know... why to get immunisation..... how to get immunisation... or where to get immunisation

Are any of these cards a problem for mothers in your community?

Are there any problems that aren’t illustrated on the cards?

What is the biggest problem? Why?

What cards aren’t problems?

Could you arrange them from the ‘biggest problem’ to the ‘smallest problem’?

Could you explain why you have arranged them like that?

**8. Recommendations**

Can you think of any ways that immunisation can be improved in this community?^[[1]](#footnote-1)^

Conclusion

Thank you for your time, it is much appreciated.

The information you have given me is very helpful

Do you have any questions? Would you like to know anything further regarding immunisations or my research?

# Appendix 2: Mothers IDI Guide

The Determinants of Immunisation Coverage in Five Rural

Villages of Sangthong District, Lao People’s Democratic Republic

Thank you for agreeing to participate in this Interview.

Do you have any questions before we start?

**Researcher Observations:**

|  |
| --- |

**Part 1: Warm up exercises**

We are just going to play a couple games to start with…

**Exercise 1:**

On these three picture cards I have some vaccines.

- This first one is polio vaccine, which you can see is given to the child orally via drops.
- The second is BCG vaccine which you can see from the picture it leaves a small scar on the child’s upper arm.
- The third is measles vaccine which is given by an injection in the arm.

Now, I have three more cards. But these cards are pictures of three diseases.

- As you can see from the picture, this first one makes you spotty- like a rash
- The second disease can make you cough a lot and have bad lungs
- The third disease can make you physically disabled, like this picture shows the cartoon is on crutches

I want you to try and match the vaccine with the disease is combats.

|  | Yes- matched correctly | No- didn’t match correctly |
| --- | --- | --- |
| Polio |  |  |
| Measles |  |  |
| BCG |  |  |

Table: Results of Exercise 1

**Exercise 2:**

Great, well done on the first exercise. Can you now tell me what age you think each of these three vaccines (polio, BCG and Measles) are given to a child? Doesn’t matter if you don’t know – just have a go!

| **Immunisation** | **Age mother thinks it should be given** |
| --- | --- |
| Polio |  |
| Measles |  |
| BCG |  |

Table: Results of Exercise 2

**Part 2: Socio-demographic features of mother**

| Age |  |
| --- | --- |
| Location from nearest HC |  |
| Method of transport to HC |  |
| Ethnicity |  |

**Part 3: Socio demographic features of each child of mother**

|  | Child 1 | Child 2 | Child 3 |
| --- | --- | --- | --- |
| Age |  |  |  |
| Sex |  |  |  |

**Part 4:** **Vaccination Card**

Do you keep a vaccination card for your child/children?

1. YES
2. NO

Do you have the vaccination card on you at the moment?

1. Yes, present - (record coverage in table below)
2. No, not present (check for BCG scar use cues for mothers recall)
3. Lost or unavailable

| **Vaccination** | **Given** | **Not given** |
| --- | --- | --- |
| BCG (birth) |  |  |
| DTPHep (6,10,14 wks) |  |  |
| Hep B (birth) |  |  |
| Measles (9mths) |  |  |
| OPV (6,10,14 wks) |  |  |
| Vit A (6,12,18,24) |  |  |
| **Mother Recall Notes:**   - Does your child have a small scar on their upper arm? - How many times has your child had an injection in the arm? - How many times has your child had an injection in the leg? - How many times has your child had drops given via the mouth? | | |

**Part 5: Mother’s knowledge.**

**Knowledge of immunisation of children in their village community**:

1. Why do you think the health facility wants you to have your child immunised?
2. Do you think that vaccines are effective in preventing your child from getting sick?
3. In what situations should a vaccine not be given?
4. How did you first hear about immunisation? Who gave you this information?
5. Where do you get most of your information now?
6. How are mothers alerted to the immunisation campaign?
7. How do you access immunisation for your child?

**Part 6: Attitudes toward childhood immunisation.**

**Attitude towards immunisation in general**

1. Now could you tell me a bit about what YOU think about immunisation of your child?
2. How important is it to you to get all the recommended immunisations for your child?
3. Very important
4. Important
5. You don’t really mind if they receive the immunisations or not
6. You do not want your child to receive immunisations at all
7. Do you think other mothers in your village have the same views you do?

**Attitude towards the way immunisation is delivered in their village.**

1. How well do you think immunisation is delivered to the children in your village?

(Ask the mother to choose)

GOOD

AVERAGE

POOR

1. Why do you think it is good / average / poor?
2. What do you think are the reasons some children receive very few?
3. What do you think are they reasons some children receive all their vaccinations?

**Part 7: Mother Practices and barriers to immunisation**

Now, we have another game to play. Here on these cards we have some pictures of things that may stop a child from getting immunised.

Before I get you to play the game, I will explain the 6 cards:

- This first card is TIME. It’s to do with mothers having enough time to get the immunisation.
- The second card is DISTANCE: as you can see the health facility in this picture is far from the village, and it is hard for the mother to get the immunisation
- The third card is AFRAID: mothers may be scared of immunisation hurting their baby, fever or side effects
- The fourth card is to do with HCWs: there may not be enough to give the immunisation, or mothers may be scared of HCWs. OR maybe HCWs don’t give mothers enough information about immunisation
- The fifth card is NO SERVICE: meaning that immunisations are not always available to mothers
- The sixth card is POOR KNOWLEDGE. Maybe the mothers don’t know... why to get immunisation..... how to get immunisation... or where to get immunisation

Are any of these cards a problem you accessing immunisation?

Are any of these cards a problem for other mothers in your village?

Are there any problems that aren’t illustrated on the cards?

What is the biggest problem (for you / mother mothers)? Why?

What cards aren’t problems (for you / other mothers)? Why?

Could you arrange them from the ‘biggest problem’ to the ‘smallest problem’?

Could you explain why you have arranged them like that?

**Part 8: Recommendations**

1. Do you have any ideas on how these obstacles you just mentioned could be overcome?
2. How else do you think immunisation services in your village could be improved?

(Using the information from the previous GAME in part 7, explore what mothers think is the best way that immunisation services could be improved in their community)

So, you ranked XXXX as the most important factor inhibiting children from receiving a vaccine, what do you think could be done to improve this situation?

**Conclusion**

Thank you for your time, it is much appreciated.

The information you have given me is very helpful

Do you have any questions? Would you like to know anything further regarding immunisations or my research?^[[2]](#footnote-2)^

# Appendix 3: Health Audit and Interview

The Determinants of Immunisation Coverage in Five Rural

Villages of Sangthong District, Lao People’s Democratic Republic

To be conducted at the health centre:

**General Observations:**

| Date: |  |
| --- | --- |
| Time: |  |
| Facility Location: | From the district hospital-  From the villages it serves- |
| Opening Times: |  |

**Information to seek from staff at health centre:**

Staff:

| Staff employed in total: |  |
| --- | --- |
| Staff employed at any one time: |  |
| Highest qualification of staff: |  |
| Staff training practices |  |

EPI Vaccination programs:

| When was the last EPI campaign run?  When is the next EPI campaign scheduled? |  |
| --- | --- |
| How is the vaccination programs promoted so communities are aware? |  |

Immunisation Stock:

Stock present at the facility at time of Audit:

| Stock | Present? (tick)  YES NO | | If yes, is this the expected amount to be present at this time?  YES NO | |
| --- | --- | --- | --- | --- |
| Vaccines |  |  |  |  |
| Auto disposable syringes |  |  |  |  |
| Sterilized wipes |  |  |  |  |
| Rubber gloves |  |  |  |  |

Maintenance / replacement of stock:

| Is there a system for maintenance and replacement of stock in place?  If yes, does this system work well? |  |
| --- | --- |
| If yes, who is responsible for monitoring the stock and reordering? |  |
| How frequently is stock delivered? |  |
| When is the next scheduled delivery?  *Note the date of audit in relation to routine delivery of supplies |  |
| Where does the stock come from? |  |
| How is stock delivered? |  |
| What is the time delay between reordering and receiving stock? |  |
| Periods of outages:  In the last three months was there a stock out for three days or more? (Y/N)  * if possible verify with stock records to look for any periods of outages, track this down to the district and health centre level |  |
| Details of periods of outages: what stock component was low in each instance? |  |
| What caused this stock component to be low / out? |  |

Immunisation cold chain equipment and system**:**

| **Equipment** | **Yes, present:**  (tick) | **No, not present:**  (tick) | **If present, is it in working order: YES** (tick) | **If present, is it in working order?**  **NO** (tick) |
| --- | --- | --- | --- | --- |
| Refrigerators at facility |  |  |  |  |
| If outreach services are conducted from the facility:  Refrigeration equipment for outreach |  |  |  |  |

| If the village immunisation is provided by outreach services, what cold chain equipment systems are put in place for this?  (Cool boxes? Cool packs?) |  |
| --- | --- |

Wastage / rubbish disposal:

| Is there any wastage of vaccine stock? |  |
| --- | --- |
| What system is used for disposal of waste? |  |
| How are sharps disposed of? |  |

^[[3]](#footnote-3)^

# Appendix 4: IDI ‘Barriers to Immunisation’ Game Sample Cards

The ’barriers to immunisation’ game was used in both mothers (Appendix 6) and HCW’s IDI’s (Appendix 5). Below are samples of the six cards used to explore barriers to immunisation.

(Please refer to IDI guides for a description of how the game was conducted)

| 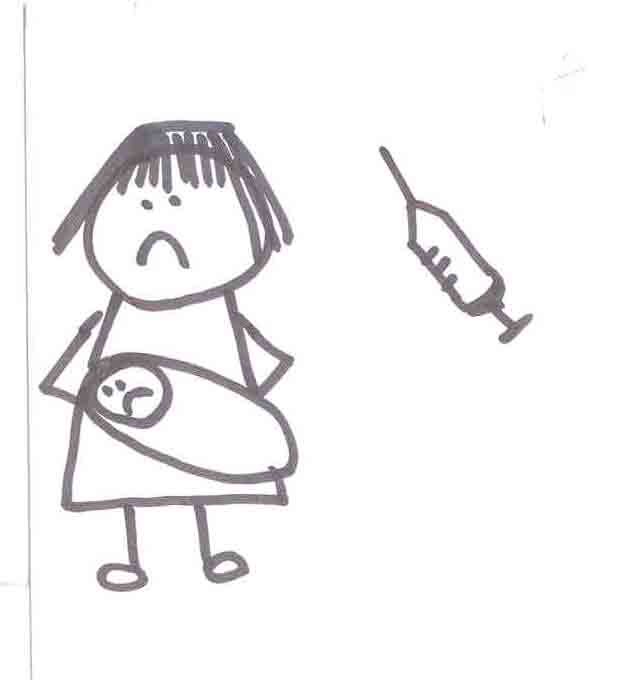 | 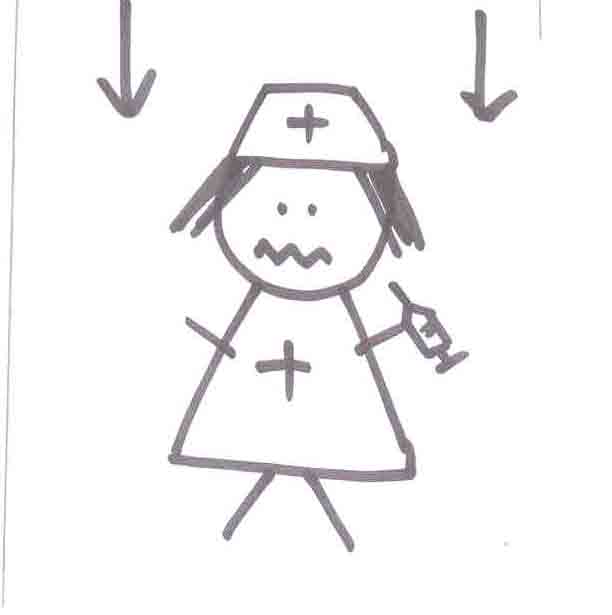 |
| --- | --- |
| 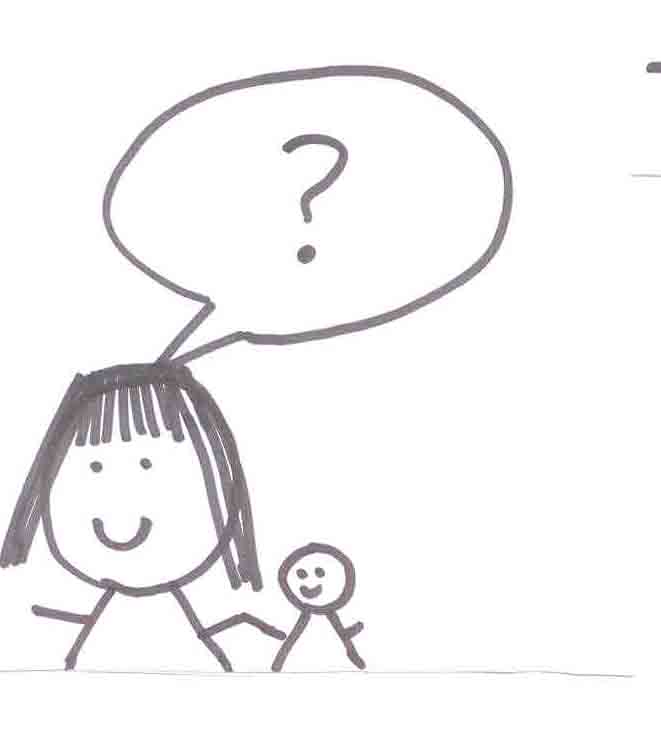 | 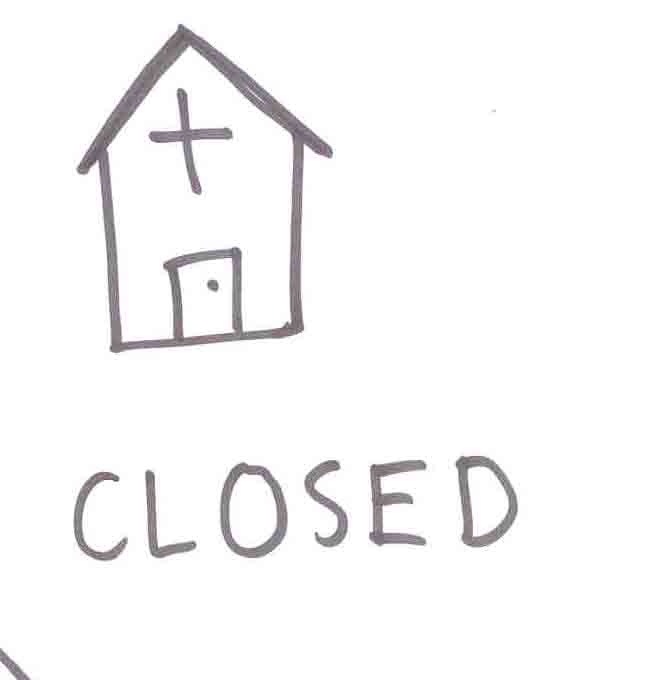 |

| 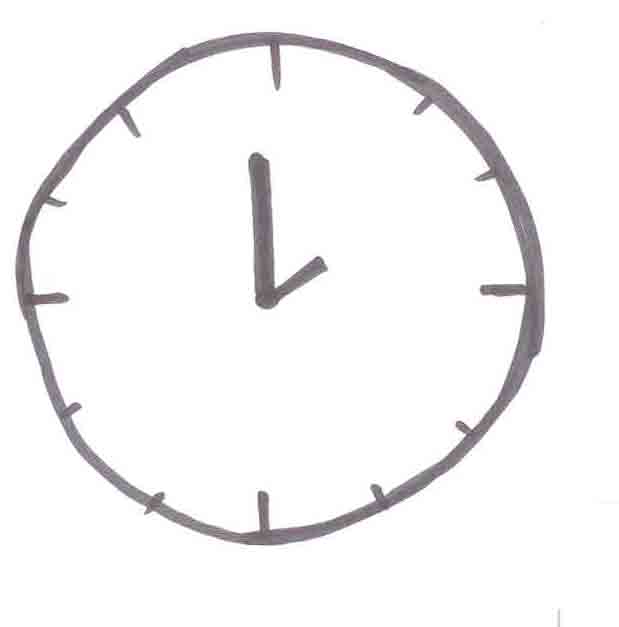 | 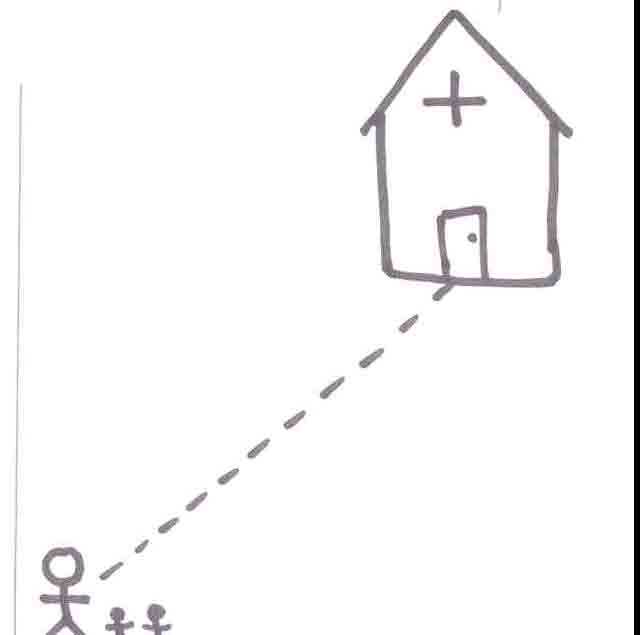 |
| --- | --- |

# Appendix 5: Mother IDI Exercise One ‘matching vaccines with diseases’ picture card samples

Please refer to part 1 mothers IDI guide (Appendix 6) for description of exercise

| CARD: POLIO VACCINE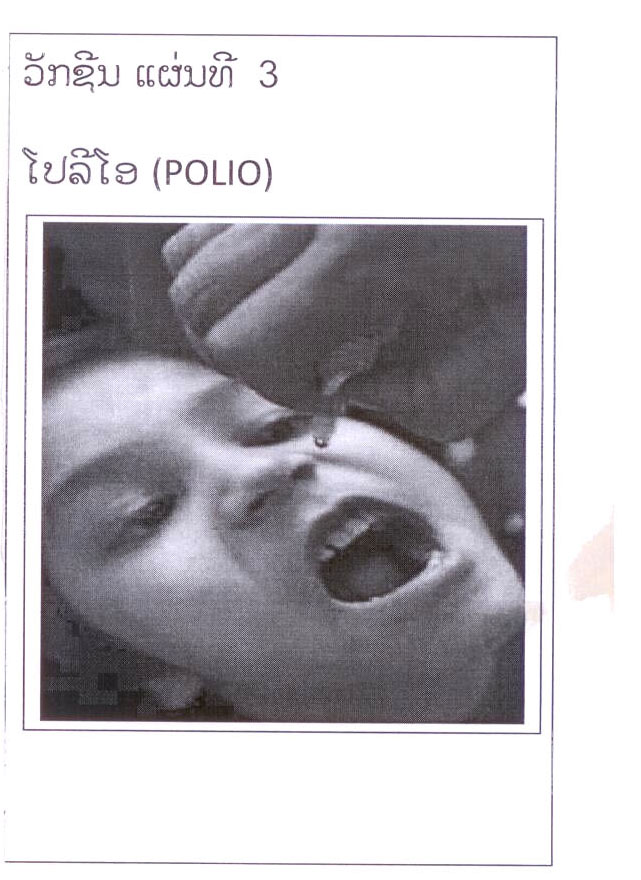 | CARD: BCG VACCINE  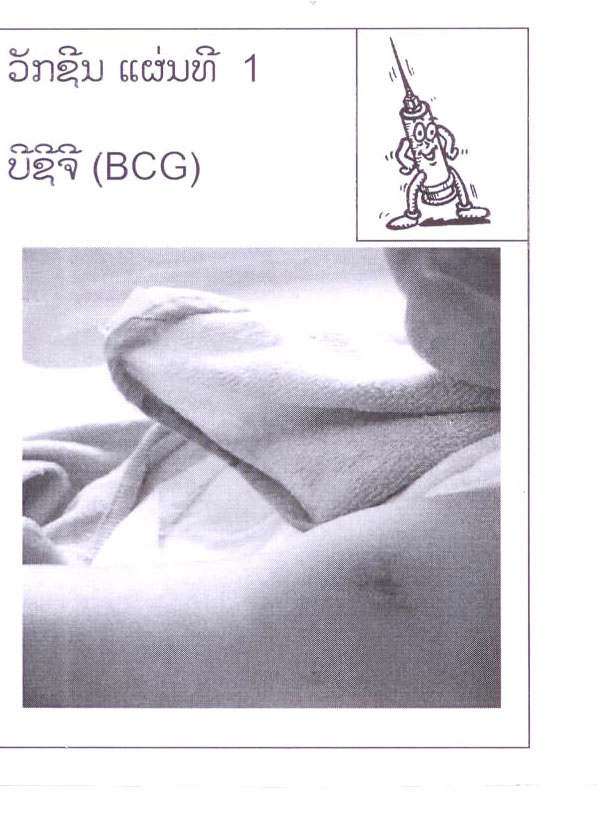 |
| --- | --- |
| CARD: MEASLES VACCINE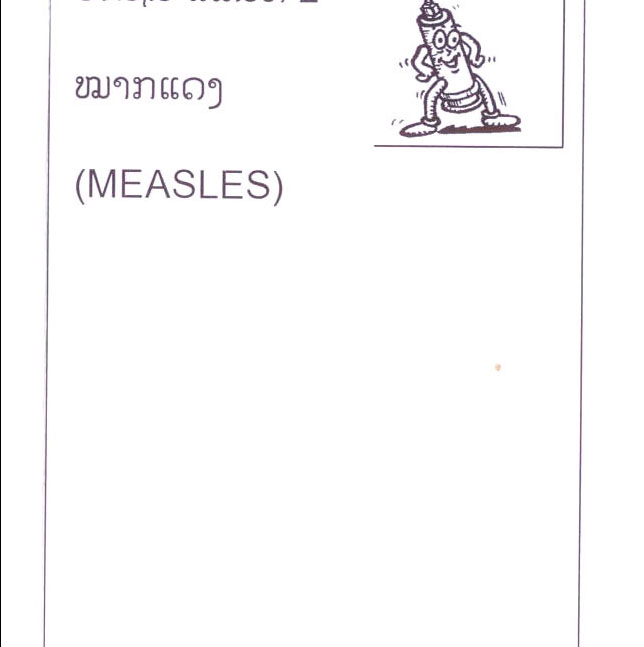 | CARD: MEASLES DISEASE 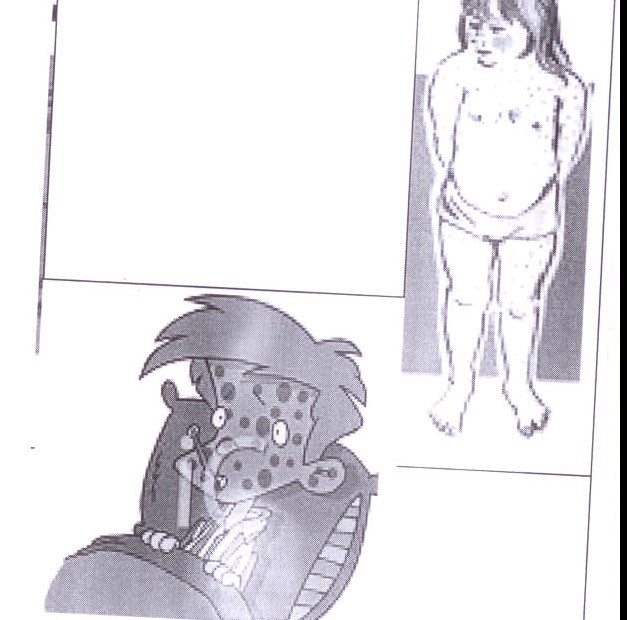 |

| CARD: TUBERCULOSIS DISEASE  **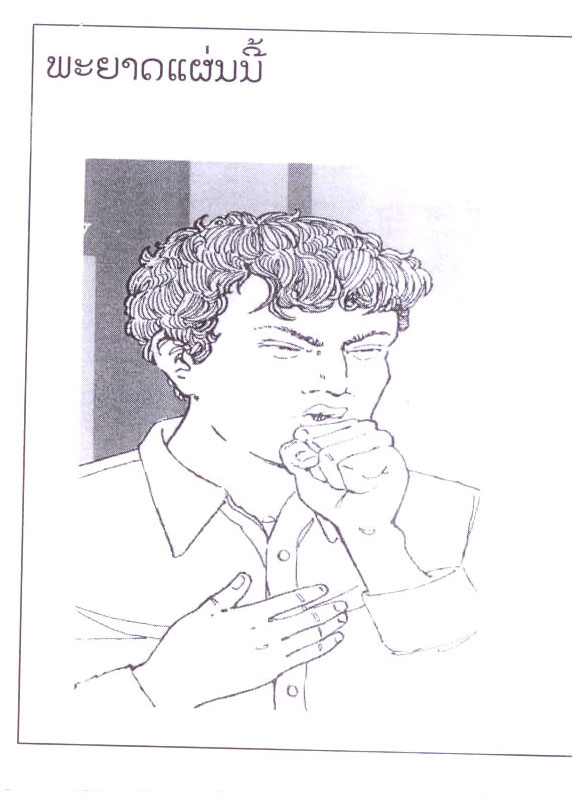** | CARD: POLIO DISEASE 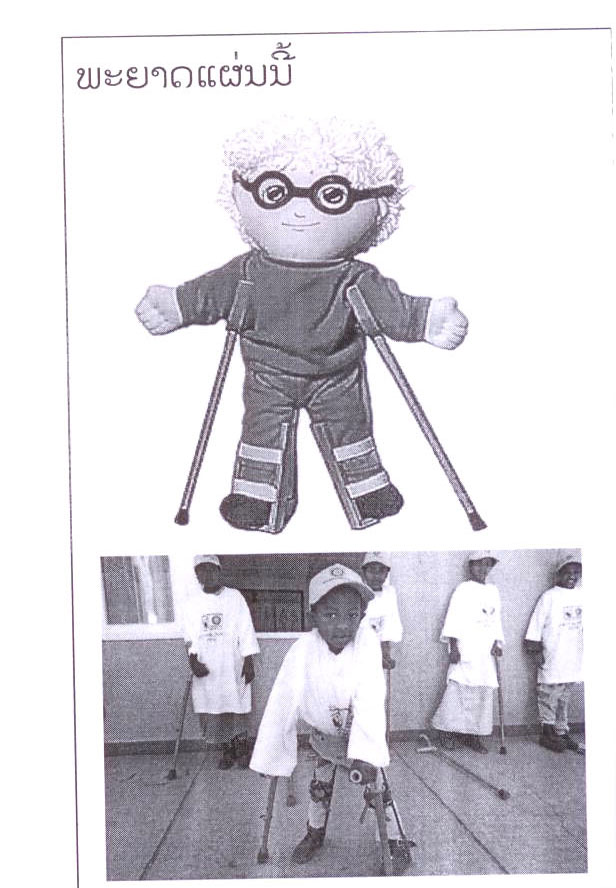 |
| --- | --- |

1. [↑](#footnote-ref-1)
2. [↑](#footnote-ref-2)
3. [↑](#footnote-ref-3)
